# THAT'S WHAT FRIENDS ARE FOR: SOCIAL CAPITAL, DEVELOPMENTAL DISABILITY, AND THE LIFE COURSE

**DOI:** 10.1093/geroni/igac059.311

**Published:** 2022-12-20

**Authors:** Jessica Hoyle, Jan Warren-Findlow, Lauren Wallace, James Laditka, Sarah Laditka

**Affiliations:** University of North Carolina at Charlotte, Charlotte, North Carolina, United States; University of North Carolina at Charlotte, Charlotte, North Carolina, United States; University of North Carolina at Charlotte, Charlotte, North Carolina, United States; University of North Carolina at Charlotte, Charlotte, North Carolina, United States; University of North Carolina at Charlotte, Charlotte, North Carolina, United States

## Abstract

Social capital, resources from reciprocal relationships, helps us get by as we age. People with developmental disability have service needs that persist across the life course, which social capital can help address. However, social structures, communication problems and smaller social networks limit social capital for some people with developmental disability. We studied how researchers conceptualize, measure, and apply social capital to developmental disability research throughout the life course, reviewing peer-reviewed articles across 5 disciplines, from 2000 through February 2022. Of 673 studies, 71 met criteria. Studies used a common definition of social capital but no common measures. Fourteen studies focused on parents or other caregivers. Few included older adults with developmental disability as research participants. Results indicate a need to better understand social capital in the lives of people with developmental disability and how social capital resources can support and improve lives of people with developmental disability as they age.

